# The ambiguous role of mannose-binding lectin (MBL) in human immunity

**DOI:** 10.1515/med-2021-0239

**Published:** 2021-02-17

**Authors:** Namarta Kalia, Jatinder Singh, Manpreet Kaur

**Affiliations:** Department of Molecular Biology and Biochemistry, Guru Nanak Dev University, Amritsar, India; Department of Human Genetics, Guru Nanak Dev University, Amritsar, India; Department of Biological Sciences, George Washington University, Washington, DC 20052, USA

**Keywords:** infectious diseases, autoimmune diseases, single nucleotide polymorphisms, functional SNPs, 5′ near gene, 3′UTR, variants, phagocytosis and MBL patents

## Abstract

Mannose-binding lectin (MBL) and lectin complement pathway have become targets of increasing clinical interest. Many aspects of MBL have been recently explored, including the structural properties that allow it to distinguish self from non-self/altered-self structures. Experimental evidences have declared the additional 5′- and 3′-variants that in amalgamation with well-known secretor polymorphisms change MBL function and concentration. Moreover, the current review highlights the differential behavior of MBL on exposure with extra/intracellular pathogens and in autoimmune diseases, stressing the fact that “high MBL levels can increase diseases susceptibility,” a paradox that needs justification. Attributable to these discrepancies, no absolute level of MBL deficiency could be defined so far and thus must be interpreted for specific diseases through case–control population-specific designs. Overall, it is evident that further research is needed about MBL and the lectin pathway of complement. Particularly, the transformative role of MBL over evolution is of interest and its role with regard to pathogenesis of different diseases and potential therapeutic targets within the respective pathways should be further explored. Apart from this, it is necessary to adopt an extensive locus-wide methodology to apprehend the clinical significance of *MBL2* polymorphisms in a variety of infectious diseases by the future studies.

## 1 Introduction

Pathogens’ identification by the innate immune system is facilitated by the germ line-encoded host factors [1]. Pattern recognition receptors (PRRs) exemplify these factors, which recognize the conserved bio-molecular structures on the pathogens’ surface, designated as pathogen-associated molecular patterns (PAMPs). The ability to recognize PAMPs and differentiate the self from non-self/altered-self structures is the characteristic and fundamental definition of PRRs. This PRR–PAMP interaction further leads to the generation of immune responses against these pathogens, eventually leading to their clearance from the host [1]. C-type lectin receptors (CLRs) are the best emerging PRRs that are gaining attention because of their numerous functions. These multifunctional properties of CLRs include endocytosis, phagocytosis, complement activation, oxidative burst, immuno-modulation by activating pro-inflammatory and anti-inflammatory responses, immune cells’ recruitment, extravasations, linking innate with adaptive immunity, controlling adaptive immune responses, regulating and collaborating with other PRRs for the optimal immune response generation, clearance of altered self-cells, and so on [2].

CLRs are the collection of asymmetric molecules possessing one or more preserved structures, referred as carbohydrate-recognition domains (CRDs), which bind to the sugars in a Ca^2+^-dependent manner [3]. On the basis of their molecular structure, these Ca^2+^-dependent proteins are of two types, such as transmembrane CLRs and soluble CLR. The transmembrane CLRs are further divided into two categories, i.e., type I and II transmembrane proteins based on the number of CRD regions and N-terminal location. The soluble CLR has an oligomeric protein structure with multiple CRDs that binds to the repetitive carbohydrate structural arrangement present on the pathogens’ surface. This soluble CLR includes a liver-based mannose-binding lectin (MBL) protein, which is a significant constituent of the human innate immunity. However, its elevated levels in the liver cirrhosis patients suggested its extra-hepatic synthesis found in the small intestine, testis tissue, and restricted areas of the gastrointestinal and reproductive tracts [4]. MBL binds to the mannose-rich PAMPs with subsequent clearing of pathogens by complement activation and phagocytosis [5].

The genetic polymorphisms of MBL and its varying serum levels are the hot topics of research in many clinical studies. However, the discrepancies observed in describing the normal and abnormal MBL levels could not provide a precision regarding the MBL’s role in health and disease. Therefore, in the current article, an effort has been made to examine the potential clarifications for the so far muddled and ambiguous discoveries reported on MBL’s function in host immunity. It first outlines the role of MBL as a prototypical PRR and the associated roles it plays in human immunity. Further, it explains and strengthens the need of more extensive locus-wide approach by future association studies to apprehend the clinical significance of *MBL2* polymorphisms, apart from the standard ones, in a variety of infectious diseases. Finally, we discussed MBL as a friend and foe in different diseases, a paradox that needs to be resolved.

## 2 MBL structure

**Figure 1:**
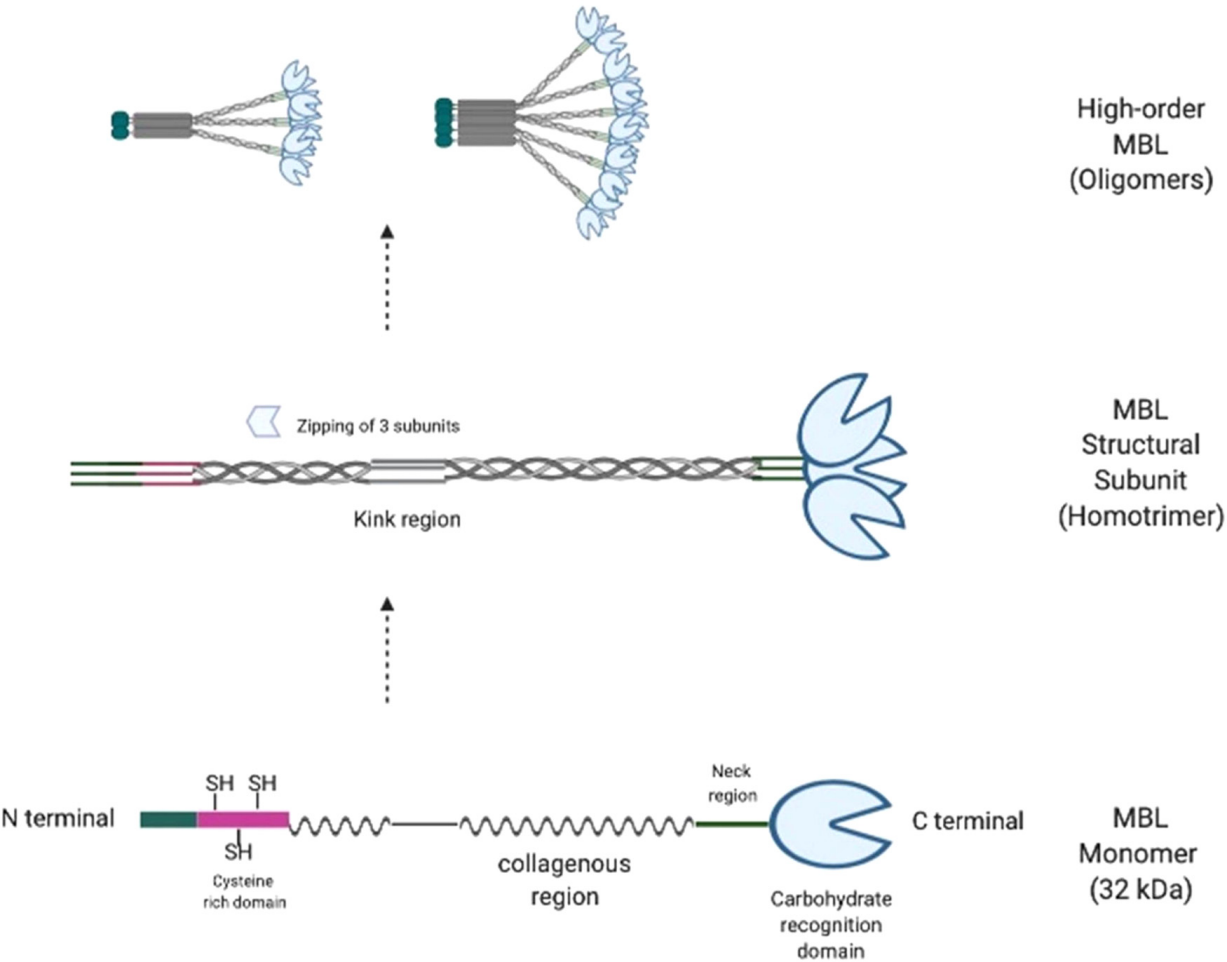
Structure and assembly of MBL (modified from ref. [113]).

The functional MBL is an extracellular circulatory protein, which is mainly expressed and produced by the liver cells. It is a bouquet-like complex that ranges from minimum two to maximum six sets of homo-trimers formed of monomers of 32 kDa protein as shown in Figure 1 [6]. This 32 kDa MBL monomer is a protein of 248 amino acids, translated from *MBL2* gene mapped to 10q11.2-q21. The monomer constitutes cysteine-rich domain, 18–20 tandem repeats of Gly-Xaa-Yaa in a collagen-like region, α-helical neck region, and a CRD from N → C terminal [7]. Owing to the triple helix nature of collagen, three monomers present with their collagen-like region in close proximity creases toward N-terminal resulting tri-meric structural subunit (the homo-trimer) with triple helix and three CRDs at C-terminal [8]. The inter-chain disulfide bonds linking tri-meric subunits further form and stabilize the high order functional oligomers found predominantly in the extracellular fluid [9]. These high order oligomers consist of multiple CRDs that allow concurrent binding of functional MBL with numerous repetitive PAMPs present on the pathogens’ surface ensuring high avidity comparative to the low binding affinity (10^−3^ M) of individual CRD–PAMP contact [10].

## 3 MBL, an ideal PRR

Structural studies revealed low affinity (10^−3^ M) interaction between individual CRD and PAMP residues. However, binding of MBL oligomer to pathogen as a whole has been shown of high affinity (10 × 10^10^ M) [11]. For these high affinity interactions, it is necessary that the ligand makes a proper geometry by spanning 45 Å in three dimensions of MBL, which is only possible in the case of repetitive carbohydrate structural patterns mainly found on the pathogen surfaces, but hidden inside the self-glycoproteins [12]. The sugar specificity is secured within the Glu-Pro-Asn amino acid sequence of CRD that favors the Ca^2+^-dependent binding with the 3′- and 4′-hydroxyl groups of carbohydrates including mannose, *N*-acetyl-d-glucosamine, glucose, fucose and their derivatives, but do not allow binding to galactose and the terminal sialic acid of oligosaccharide chains present on the host cell surface [13,14]. In addition, abnormal glycosylation occurs in the cancerous cells because of oncogenic transformation that leads to the exposure of sufficient sugar ligands in fitting pattern, allowing their recognition and binding by MBL [15]. Besides this, MBL recognizes apoptotic cells with exposed hidden repetitive sugar pattern [16]. Thus, MBL can also recognize altered self-structure and mediates cytotoxicity and clearance by phagocytosis. Therefore, three structural properties, i.e., (i) blocking of oligosaccharide chains with terminal sialic acid residues, (ii) inadequately exposed sugar ligands to allow precise binding configuration, and (iii) hidden repetitive sugar pattern inside glycoproteins, allow MBL to distinguish self from the non-self and altered-self structures, making it an ideal PRR. This prototypical PRR has been shown to bind diverse range of fungal, bacterial, parasitic, and viral species, thereby generating required immune responses [17,18,19,20,21,22].

## 4 The receptor-mediated effector functions of MBL

The receptor-mediated effector functions of MBL include complement (C) system activation, direct opsono-phagocytosis, MBL-dependent cell-mediated cytotoxicity, apoptosis, pro-inflammatory cytokines release, and ROS production. These are described herewith.

### 4.1 Activation of complement (C) system

The C system involves the interactions of distinct plasma proteins that opsonize the pathogens and initiates a series of inflammatory reactions for the ultimate clearance of pathogens [23]. Activation of C system on the pathogen surfaces involves three pathways, i.e., the classical, lectin, and alternative, involving different initiatory molecules that eventually converge to produce similar molecules [23]. MBL binding to the pathogen surface activates the so-called MBL-mediated lectin complement pathway. There is a family of serine proteases that bind to MBL and get activated when the complex is associated with the pathogen. These MBL-associated serine proteases (MASPs) include MASP-1 to -3 along with the truncated version of MASP-2 currently named as MAP-2 (also known as Map19 or sMAP), of which MASP-2 has been shown to play an important role in the C system activation [23,24,25,26,27,28]. Following activation, MASP-2 results in the consecutive cleavage of C4 and C2, generating C4b and C2a fragments that interact with each other to form C3 convertase (C4bC2a complex) that will eventually converge with the other C3 convertases produced from both the alternative and the classical pathways. These C3 convertases then start the C cascade activity generating the terminal components of C system that gather to form membrane attack complex and damage the pathogen. Lectin pathway also generates opsonic C3b and other C fragments, which further enhance the phagocytosis as well as activate and infiltrate the additional phagocytes to the spot of C activation.

### 4.2 Direct opsonophagocytosis by MBL

Besides causing indirect opsonization because of the C components (opsonins) generated as a result of MBL pathway, MBL can directly act as an opsonin, independent of C system activation [29,30,31]. This direct opsonophagocytosis by MBL has been shown to be facilitated by the receptors present on the surface of phagocytic cells (monocytes and neutrophils) [32,33]. Other than this, several other intracellular and extracellular receptors have been defined in the literature, which directly bind to the MBL, although their function is not completely understood. These receptors include cC1qR (calreticulin), C1qRp, complement receptor 1 (CR1), alpha-2-beta-1 integrin, and α-2-macroglobulin receptor (CD91) [34,35,36,37,38,39,40]. This imperative property explains why the low levels of MBL has ultimately been suggested to be responsible for opsonic defects found in the children with recurrent infections, which was earlier thought to be occurring because of the deficiency of other opsonins [41,42].

### 4.3 MBL-dependent cell-mediated cytotoxicity

Researchers have detected MBL binding to the aberrant carbohydrate structures expressed by the cancerous cells and mediating cytotoxic activity, which was referred as MBL-dependent cell-mediated cytotoxicity [43,44]. It is important to note that this activity was shown by the mutated MBL protein with no opsonic and complement activation activity, suggesting the un-described cytotoxic role of MBL, whose relative importance in tumor immunology is currently not known.

### 4.4 Role in apoptosis

As mentioned above, MBL can directly bind to the apoptotic cells with exposed repetitive sugar pattern, thereby permitting their recognition and the ultimate clearance of apoptotic cells by phagocytes [16,45]. Low MBL levels have been suggested to be associated with defective apoptotic cell clearance [46].

### 4.5 Pro-inflammatory cytokine release

MBL has been shown to trigger the release of pro-inflammatory cytokines from the monocytes [47,48]. A study by Jack et al. [49] depicted the elevated production of the pro-inflammatory cytokines including IL-6, IL-1β, and TNF-α from the monocytes at low MBL levels (4 μg/mL). However, the inflammatory production reduces at high MBL concentrations, suggesting the complex role of MBL in modulating the release of pro-inflammatory cytokines with unexplained mechanisms.

### 4.6 Reactive oxygen species (ROS) production

Other than the above-mentioned functions, MBL mediates ROS production including superoxide and H_2_O_2_ from human neutrophils [30,33,50].

Thus, MBL is one of the best examples of a single molecule with multiple functions ranging from an ideal PRR, activation of C system, direct and indirect opsonization, altered self-cells clearance, pro-inflammatory cytokines release to ROS production. These important functional activities explain why the defect in MBL has been shown to be associated with different infectious diseases such as malaria, human immunodeficiency virus (HIV) infection, leprosy, leishmaniasis, schistosomiasis, trypanosomiasis, tuberculosis, systemic lupus erythematosus, rheumatoid arthritis, recurrent vulvovaginal infections (RVVI), filariasis, etc. [51,52,53,54,55,56,57,58].

## 5 *MBL2* structure

**Figure 2:**
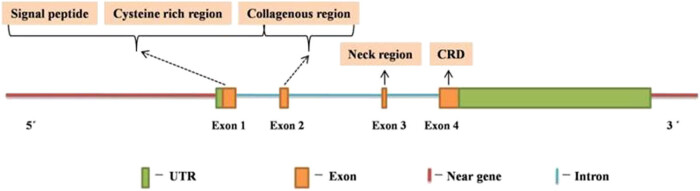
Structure of *MBL2* (modified from ref. [114]).

Humans have two MBL genes, *MBL1* and *MBL2*, of which *MBL1* is a pseudogene, leaving only one functional gene *MBL2* that encodes for MBL protein [59]. *MBL2* comprises 7,461 bases, between 5,27,65,380 and 5,27,72,841 bp region of chromosome no. 10 (10q11.2-q21) (NC_000010.11, NCBI), translating into MBL protein of 248 amino acid (NCBI accession number XP_011538118.1) via a 3,570 bp long mRNA (NCBI accession number NM_000242.21). The gene structure of *MBL2* is same as that of other eukaryotic genes with each element contributing to the specific function in gene expression. *MBL2* comprises four exons and three introns. The exons determine the structure of proteins with exon 1 encoding signal peptide, a cysteine-rich domain, and a portion of glycine-rich collagenous region. Exon 2 encodes the other portion of the collagenous domain, whereas α-helical “neck” region and CRD are encoded by exons 3 and 4, respectively (Figure 2). In addition, the promoter and 3′UTR region of the *MBL2* contain various elements that have been shown to regulate *MBL2* expression [60,61,62,63].

## 6 *MBL2* genetics (SNPs with validated functional consequences)

An acute phase protein is the commonly referred term used for MBL accredited to the elevated serum levels’ subsequent infection and under aberrant body changes [64]. However, MBL is not a typical acute phase protein, because its response is generally demonstrated by the individuals’ MBL levels and genetic variations [65,66,67]. Single nucleotide polymorphisms (SNPs) in exon 1 and promoter region of *MBL2* have been functionally characterized in regulating serum MBL (sMBL) levels. SNPs in exon 1 include rs5030737/codon 52, rs1800450/codon 54, and rs1800451/codon 57, jointly referred as *MBL2* structural variations [68]. SNPs including rs1800450 and rs1800451 involve the glycine replacement with the di-carboxylic acids. In contrast, SNP rs5030737 involves arginine replacement with cysteine in the MBL monomers’ collagenous region consequently forming the variant subunits [69,70]. These variant monomers further impede the synthesis of high order MBL oligomers because of the distortion created in the triple helix collagenous region by codon 54 and 57 variations. Besides this, the additional disulfide bonds that are formed as a result of extra cysteine residues added by codon 52 variation also impede the MBL oligomers synthesis. These structural changes dramatically lower the synthesis, circulating levels, and functional activity of high order MBL oligomers [69,70,71,72,73,74]. Apart from these structural variations, promoter polymorphisms including rs11003125; L/H, rs7096206; Y/X, and rs7095891; P/Q regulate promoter activity of the gene subsequently modulating the MBL protein levels. From this, the biological implication of the genetic variations can be explicated in regard to the *MBL2* transcription and translation [75,76,77]. *MBL2* promoter and structural polymorphisms are in strong linkage disequilibrium (LD) forming the seven haplotypes, universally referred as secretor haplotypes involving LYPA, HYPA, LXPA, LYQA, LYPB, LYQC, and HYPD. The haplotypes involving HYPA, LYQA, and LYPA confer the high sMBL levels, whereas haplotypes involving LXPA, LYPB, LYQC, and HYPD confer the low sMBL levels [78]. Apart from the seven standard ones, the other haplotypes counting HYQA, HXPA, LXPB, LYQB, and LYPD have been reported by different population-specific studies [79,80,81]. This suggests the emergence of novel haplotypes besides the standard ones, owing to the heterogeneity found in the genetic pool of the various populations and the observed discerning advantage because of the ecological pressures like infections or demographics [82].

However, there are studies that did not find the complete correlation of sMBL levels with the secretor haplotypes signifying the existence of other *MBL2* regulating variants [20,78,83]. To reveal this, Bernig’s group in 2004 analyzed the complete *MBL2* gene of 10.0 kbp by re-sequencing it and found a great degree of heterozygosity [84]. They further found that the LD pattern of complete *MBL2* uncovers a plausible recombination hotspot of 1.6 kbp that lies in the coding region of exon 4 in the 3′ end of the gene. This hotspot divides the whole *MBL2* into two separate haplotype blocks in which the markers within each block are in linkage with each other, while the markers of one block are not in linkage with the markers of other. They named these blocks as the 5′ and 3′ haplotype blocks or simply as 5′ and 3′ blocks of *MBL2* gene [84]. Moreover, the structural SNPs of exon 1 along with the upstream promoter polymorphisms that form the “secretor haplotypes” were found to lie in the 5′ block and are part of its extended haplotypes, indicating the possibility of additional linked 5′ variants with the possible functional implications. To investigate this, Bernig’s group in another study analyzed both *MBL2* blocks in the unrelated Dutch Caucasians [61]. The results of this study suggested that the other variants in 5′ as well as 3′ haplotype block may modify the protein functionality and serum levels (Table 1). To further confirm these findings, the same group presented the preliminary evidence authenticating that 3′UTR variants of *MBL2* manipulate the MBL concentration and also interact with the haplotypes of 5′ block that might increase the risk of breast cancer in the African-American women [62]. Other studies by different groups also found that the combined 5′ and 3′ blocks of *MBL2* alter sMBL levels and increase risk of postoperative myocardial infarction, colon cancer, and RVVIs in White-Americans, African-Americans, and Indians, respectively [54,55,63,85]. Because of these observed associations, Zanetti et al. [63] functionally characterized the 3′UTR haplotypes of 3′ block and found that the SNP rs10082466 in the 3′UTR haplotype generates a binding site for miR-27a with full complementarity to its seed region. This binding has been shown to hasten the mRNA degradation by microRNAs (miRNAs) resulting in reduced protein translation as depicted by a significant decrease found in the normalized luciferase activity of *MBL2* [63]. Overall, this implicates the necessity to adopt an extensive locus-wide methodology by the future association studies to apprehend the clinical significance of *MBL2* polymorphisms in a variety of infectious diseases.

**Table 1:** Functionally characterized polymorphisms of *MBL2*

dbSNP identifier^a^	SNP position (5′ → 3′)^b^	Chromosome position^b^	Secretor^c^	Region	Nucleotide change	Effect	Characterized function	References
rs11003125	−618	5,27,72,254	L/H (aka −550)	5′ near gene	C/G	Variation in the promoter activity of *MBL2*	Alter transcriptional levels	[75,76,77]
rs7096206	−289	5,27,71,925	Y/X (aka −221)	5′ near gene	G/C			
rs7095891	−65	5,27,71,701	P/Q (aka +4)	5′ near gene	C/T			
rs5030737	+152	5,27,71,482	A/D (codon 52)	Exon 1	C/T	Distortion in the triple helix collagenous region	Dramatically reduce the formation of functional higher order oligomers	[69,70,71,72,73,74]
rs1800450	+161	5,27,71,475	A/B (codon 54)	Exon 1	G/A			
rs1800451	+170	5,27,71,466	A/C (codon 57)	Exon 1	G/A	Formation of adventitious disulfide bonds		
rs10082466	+4,455	5,27,66,862	—	3′UTR	A/G	Generates a binding site for miR-27a	Hasten mRNA degradation by miR-27a with reduced protein translation	[63]

a NCBI dbSNP database (http://www.ncbi.nlm.nih.gov/SNP).

b NCBI contig accession number NT_030059.14 (NCBI build 142, locus ID 4153).

c Designation extensively used in the literature is also followed in the present study, and these markers form the “secretor haplotypes.”

## 7 MBL in different diseases and MBL paradox

The important functions of MBL in immune defense led to the following implicit assumption: “High serum MBL (sMBL) levels provide protection and low levels risk to the diseases.” This assumption was initially documented in 1989, when the defect in opsonization because of MBL deficiency (i.e., low sMBL levels) was recognized as the reason of frequent inexplicable infections in children [42]. After this, the number of pediatric population-specific studies started emphasizing the importance of nonspecific innate immunity provided by MBL in the early life. The later was considered as the substitute for the defense offered by the adaptive immunity, which is suggested to be undeveloped in infants. Nonetheless, the studies based on the adults suggested the role of MBL throughout one’s life, emphasizing its role as a first line of defense against pathogens till the adaptive immunity starts responding [86]. A synopsis involving specific examples covering the mushrooming literature regarding MBL in different diseases is discussed below:

### 7.1 MBL in infectious diseases caused by extracellular pathogens and autoimmune diseases

Low sMBL levels (i.e., MBL deficiency) have been shown to be predisposing individuals to many infectious diseases specifically caused by the extracellular pathogens [87]. One of the earliest evidences in this regard was observed in the AIDS patients with *MBL2* structural variant alleles. These patients were found to have a low survival rate attributed to their increased susceptibility to coinfections [88]. In addition, the number of studies have shown the MBL-deficient states in patients with hepatitis B or C indicating that the low sMBL levels increase susceptibility to this disease [89,90]. The same scenario was also observed in the autoimmune diseases where MBL deficiency was shown to predispose individuals to systemic lupus erythematosus [79] and rheumatoid arthritis [91]. In general, a surplus literature can be found vis-à-vis *MBL2* genetic variations and their association with low sMBL levels in susceptibility to many diseases including tuberculosis, malaria, filariasis, systemic lupus erythematosus, trypanosomiasis, rheumatoid arthritis, HIV infection, RVVIs, and many more (as afore referred), not to mention the severe acute respiratory syndrome-coronavirus (SARS-CoV) infection.

It has been documented that MBL paucity upsurges the risk of SARS-CoV-1 infection [92] with a higher prevalence of low secretor haplotypes in SARS cases than controls. Of all the viral envelopes to which MBL can bind, both plasma-derived and recombinant human MBL have been shown to directly inhibit SARS-CoV spike (S) glycoprotein (SARS-S)-mediated viral infection [93]. Therefore, MBL binding to SARS-S may intrude the events necessary for the efficient viral entry. This further suggests a possible role of MBL in the coronavirus disease 2019 (COVID-19). In this respect, a recent study suggested that SARS-CoV-2 uses the mannose-rich S protein for binding the angiotensin-converting enzyme 2 (ACE2) receptor of host to mediate host-cell entry [94]. This provides an assumption that MBL may possibly bind and inhibit the S-ACE2 interaction in SARS-CoV-2, as it did for SARS-CoV-1. A recent pre-print by Gao et al. [95] strengthened this speculation, by suggesting the role of MASP-2-mediated complement over-activation on interaction with N protein dimers of SARS-CoV-2 that aggravates the lung injury. Moreover, a case–control study showed higher expression of MBL in the lungs of COVID-19 group than controls [96]. In contrast, comparable plasma MBL levels were found between COVID-19 cases and controls by another study [97]. These two case–control studies together provide the inclusive findings attributed to their underpower study design. Given the prevalence of this disease, it is very important that such case–control studies must be performed in the larger datasets of different populations, so that the role of MBL in COVID-19 can be elucidated. This is of utmost importance as the presence of symptomatic COVID appears to be dependent more on the host factors rather than on the pathogen itself. Therefore, the knowledge of MBL role in COVID-19 may help us devise an efficient immune-based therapy.

### 7.2 MBL in infectious diseases caused by intracellular pathogens (the paradox)

The paradox, i.e., “high MBL levels increase diseases susceptibility and low MBL levels protect,” came into light when MBL was found to have predisposing effect on the infectious diseases involving leprosy and visceral leishmaniasis, both caused by the intracellular parasites [98,99]. These patients had considerably high sMBL levels in comparison to the controls. In unison, the higher prevalence of variant alleles observed in controls than in patients suggest the advantageous role of functional MBL deficiency in these controls. This unexpected frequency was explained by the fact that intracellular parasite is dependent on the phagocytosis to invade the host cells and use opsonization by complements to enter the phagocytes through complement receptors. Therefore, the presence of low MBL levels in the controls will consequently lower the probability of parasitization because of the reduction in the complement-mediated phagocytosis [98]. This could be the plausible rationale behind the few exceptions that have surplus existence of low MBL phenotypes across the globe, suggesting that these geographic areas must be prone to the intracellular parasitism. Therefore, any mechanism that diminishes C system activation would be favorable conferring the selective advantage to those individuals carrying the variant alleles and low MBL levels [71,100]. The mechanism was proposed to be similar to sickle cell anemia, where the sickle cell gene was found to provide protection against malaria that ultimately leads to the selective advantage to the carriers of sickle cell allele [101]. Overall, this suggests that low sMBL levels provide protection against intracellular pathogens.

Thus, these reports mentioned above have suggested the protective role played by MBL against diseases either through high levels or selectively by low levels. In contrast, there are reports that did not find any significant difference between the MBL levels of cases and controls [102,103]. Moreover, MBL-deficient individuals with no evidence of infections have also been reported, which argues against the defensive role played by the MBL [102,103]. However, these studies cannot be viewed as the evidence of no clinical applicability of sMBL levels. This is because different groups of scientific researchers have provided the evidence verifying the defensive role of MBL under a case–control design. In addition, the studies have also suggested that MBL paucity works in synergism with other humoral immunodeficiencies to cause diseases [104,105]. These are the discrepancies because of which no absolute level of MBL deficiency could be determined. Therefore, it is important that instead of generalizing the MBL’s role, an effort should be made in defining its versatile nature in different diseases.

## 8 Clinical trials and patents

The plasma-derived MBL has passed phase-I clinical trials and has successfully been used for the treatment of a 2-year-old girl with opsonic defect suffering from the devastating recurrent infections and other patients with cystic fibrosis and lung infections [106,107,108,109,110,111]. Furthermore, patents have been filed for the use of recombinant MBL for the management of melanoma cancer, lymphoma cancer, pancreatic cancer, ovarian cancer, or colorectal cancer (WO2008019322A2, US6846649B1, and WO2004026330A1). However, after some enthusiasm about MBL substitution treatment, there is a lack of well-designed large trials that could have supported the adoption of MBL supplementation. Patient numbers in the references mentioned are small, and there has been no widespread clinical use of MBL ever since. In light of this, the present literature review commands further research using larger datasets of different populations for the use of emerging MBL-supplementation as effectual treatment for different diseases in which its role has already been elucidated to authenticate the medicinal prospects of MBL.

## 9 Conclusion and future directions

MBL harbors a complex genetic system and several studies have shown the association of its variants with infectious diseases, suggesting its transformative role in innate immunity, exemplifying how polymorphisms were shaped by ecological pressure like infections and demographics [112]. Therefore, further research focused on elucidating MBL and the lectin pathway of complement with regard to the pathogenesis of different diseases and potential therapeutic targets within the respective pathways along with the exploration of the neglected area of “whether pathology due to MBL paucity entails one or more co-existing immune deficits or not” is needed as supported by different studies [68,104,105].
